# Light‐Responsive Bilayer Cell Culture Platform for Reversible Cell Guidance

**DOI:** 10.1002/smsc.202100099

**Published:** 2021-12-16

**Authors:** Mari Isomäki, Chiara Fedele, Lotta Kääriäinen, Elina Mäntylä, Soile Nymark, Teemu O. Ihalainen, Arri Priimagi

**Affiliations:** ^1^ Faculty of Engineering and Natural Sciences Tampere University Korkeakoulunkatu 3 FI‐33720 Tampere Finland; ^2^ BioMediTech and Faculty of Medicine and Health Technology Tampere University Arvo Ylpön katu 34 FI‐33520 Tampere Finland

**Keywords:** azobenzene, cell culture, epithelial cell alignment, light-responsive cell culture platform, micropatterning, surface relief gratings

## Abstract

In vivo, cells are surrounded by a constantly changing microenvironment, which regulates many cell functions such as differentiation, migration, and cell death. Stimuli‐responsive biomaterials aim to mimic this interaction between cells and extracellular matrix in vitro. However, reproducing dynamic signaling noninvasively without affecting the cell viability remains a challenge. Herein, a dynamic cell culturing platform consisting of a light‐responsive azobenzene molecular glass film and a protective polydimethylsiloxane (PDMS) coating is developed. By tuning the PDMS layer thickness, surface relief gratings (SRGs) can be efficiently photoinscribed on the platform surface. The SRGs can also be erased with light in the presence of PDMS, i.e., the topography can be reversibly photomodulated. The inscribed SRGs can guide epithelial cell orientation along the topography. The erasure parameters are targeted toward cell culturing environment, enabling experiments with live cells. Finally, the photoresponsive platform is patterned with proteins by microcontact printing, allowing its biofunctionalization and the combination of microtopography and protein patterns. This study paves the way for using reconfigurable cell culture platforms for the dynamic control of cell–material interactions. The PDMS coating has potential to protect underneath material, broadening the spectrum of possible materials for dynamic cell culture platforms.

## Graphical abstract

Herein, a dynamic cell culture platform consisting of a light‐responsive azobenzene molecular glass film and a protective polydimethylsiloxane (PDMS) coating is presented. The platform can be photopatterned and ‐erased, and the resulting surface topography can guide epithelial cell alignment. The topography can be changed in the presence of live cells and further patterned with proteins.

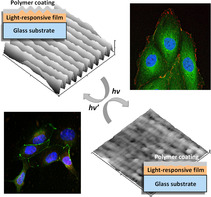

## 1 Introduction

Cells are continuously interacting with other cells and the surrounding extracellular matrix (ECM), which consists of water, polysaccharides, and many proteins.^[^ ^1^ ^]^ The biophysical and biochemical signals that originate from the ECM regulate the dynamic interplay between the cells and the microenvironment. Several cell functions, such as migration, differentiation, and death, are coregulated by the local external microenvironment.^[^ ^2^ ^]^ These functions are related to physiological processes like tissue repair and tumor growth.^[^ ^1^ ^]^ Materials designed for biomedical applications mimic this interaction, however, in cell culture applications they often lack the dynamic nature of native tissue and without the natural microenvironment some of the cell functions can be lost or altered.^[^ ^3^ ^]^ There is a growing interest in smart biomaterials that can respond to external stimuli. The possibility to dynamically change the properties of the material would create new opportunities for disease modeling in vitro.^[^ ^2^ ^]^

**Figure 1:**
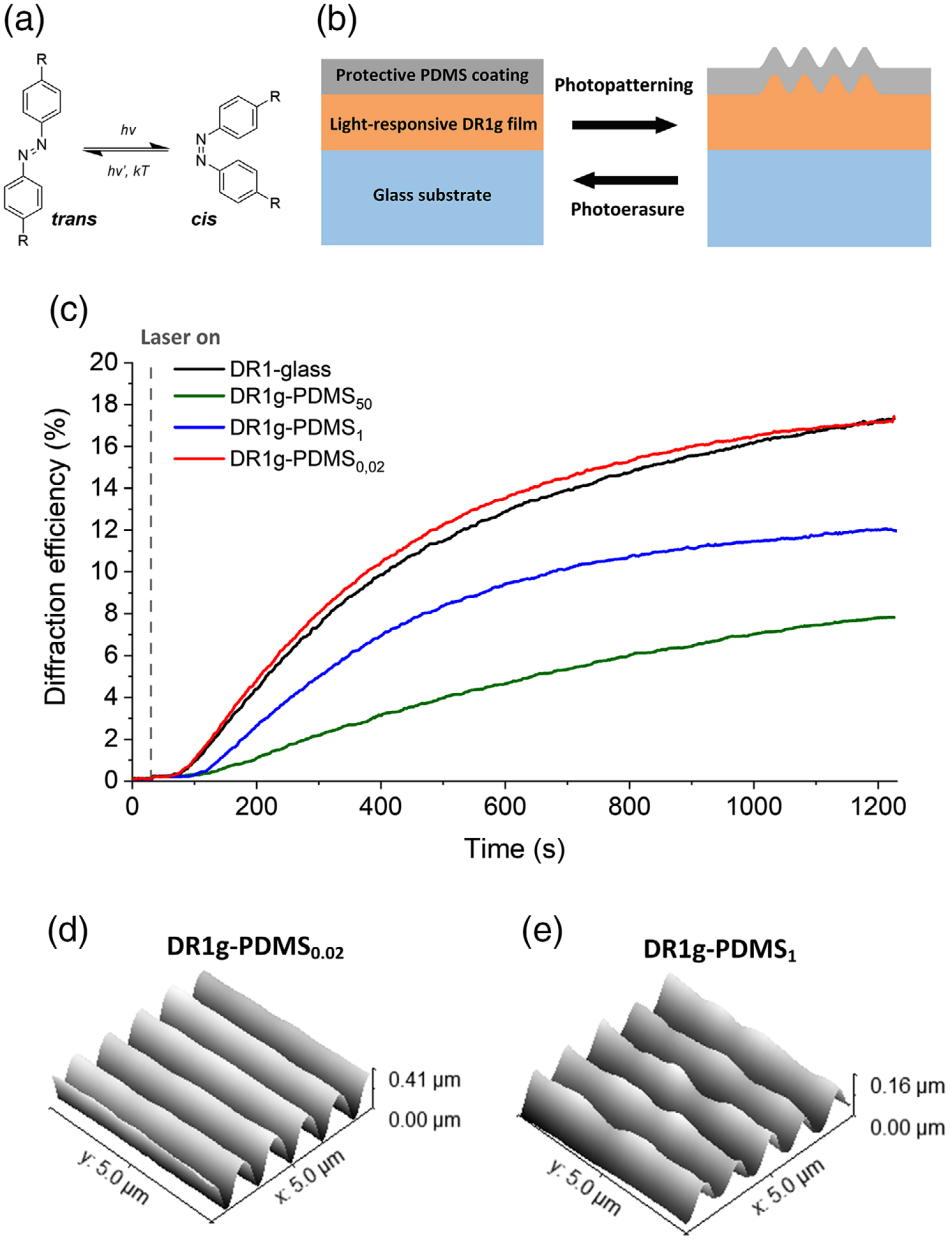
a) Photoisomerization of azobenzene between the thermodynamically stable trans and the metastable cis isomer. b) Graphical representation of the bilayer structure and the azobenzene‐driven SRG formation and erasure. c) Diffraction efficiency curves (averaged over three measurements for each curve) with different thicknesses of PDMS, during SRG inscription with intensity of 300 mWcm^−2^ (488 nm, circular polarization, probe beam wavelength 633 nm). Standard deviations for the DE values at the end of the SRG inscription are ±7% (DR1g), ±0.5% (DR1g‐PDMS_50_), ±5% (DR1g‐PDMS_1_) and ±6% (DR1g‐PDMS_0.02_). AFM images of the surface topography on d) DR1g‐PDMS_0.02_, and e) DR1g‐PDMS_1_ after SRG inscription.

Light is an attractive energy source for stimuli‐responsive biomaterials, as it is a noncontact stimulus that can be localized to a desired area noninvasively, and its properties (wavelength, intensity and polarization) can be precisely controlled.^[^ ^4^ ^]^ One of the widely used molecules for photosensitive materials is azobenzene.^[^ ^5^, ^6^ ^]^ The molecular process behind the photoinduced motions in azobenzene‐based systems is the isomerization of the azobenzene molecule between the thermodynamically stable *trans* isomer and metastable *cis* isomer (**Figure** **1**a).^[^ ^5^, ^7^ ^]^ Azobenzenes can be incorporated into polymers to create thin amorphous azopolymer films, which can be surface patterned via light‐induced mass migration. With light interference lithography, sinusoidal surface relief gratings (SRGs) can be inscribed,^[^ ^8^, ^9^ ^]^ superposed,^[^ ^10^ ^]^ erased,^[^ ^11^ ^]^ and rewritten.^[^ ^12^, ^13^ ^]^ SRGs can guide cell elongation and alignment along the microtopography and this property of azobenzene‐based materials has been recently used to guide cell adhesion, growth, collective cell migration and neuron guidance.^[^ ^14^, ^15^, ^16^, ^17^ ^]^ We envision that the reversibility of the photoinduced surface patterns will be the key element for dynamic control of biomaterials. Azobenzene‐based materials have been used, for instance, as photoswitches to control biomolecules^[^ ^18^ ^]^ and in photoactive drug delivery applications.^[^ ^19^ ^]^ Despite the great potential, their use as cell culturing platforms is still in its infancy and research related to dynamic topography change in the presence of live cells is scarce.

As azobenzene‐based materials have not been widely used as cell supporting materials, and due to the large number of different materials available, their stability in cell culture environment has not been extensively studied. For this reason, it would be highly advantageous to control the light‐responsiveness and cell–material interface independently with a separate coating. This is a common strategy to improve material biocompatibility with natural or synthetic materials.^[^ ^20^, ^21^ ^]^ Particularly, many polymers are promising for creating biocompatible surface coatings for biomedical applications, since their mechanical properties can be tuned to resemble the properties of native tissue.^[^ ^20^ ^]^ Polydimethylsiloxane (PDMS) is a commonly used silicone elastomer in medical devices and its properties, such as being chemically inert, stable, easy‐to‐use, and transparent, make it a promising candidate for biocompatible surface coatings.^[^ ^22^ ^]^ PDMS is also widely used in cellular studies, and patterned PDMS substrates have been utilized to guide cell alignment.^[^ ^23^, ^24^, ^25^ ^]^ In addition to mechanical stimuli, cells are affected by biochemical signals in natural tissue. Since cells respond to both biophysical and biochemical signals, it is advantageous for a biomaterial to have the ability to be patterned with proteins (providing localized biochemical cues), without affecting other properties of the material. The effects of either physical microtopography or protein pattern have been investigated broadly, however, the research about the combination of these two is still limited.^[^ ^26^, ^27^ ^]^ Therefore, there is still poor understanding on how these two signals are interplaying and regulating cell behavior. Polymer coatings facilitate the use of many soft lithography techniques, such as microcontact printing (μCP).^[^ ^28^ ^]^ This technique has been used to print different protein patterns on nonadhesive materials.^[^ ^29^ ^]^

Herein, we present a light‐responsive cell culture platform consisting of a thin layer of azobenzene‐based molecular glass^[^ ^30^ ^]^ with a thin PDMS coating. The underneath photosensitive film ensures light responsivity and the PDMS layer on top enables independent control of stability and cytocompatibility. We study the effect of different PDMS coatings on the SRG formation efficiency, as well as light‐driven erasure of the SRGs in the presence of PDMS. We observed that with 65 nm thick PDMS layer the SRG formation and erasure on the bilayer is still efficient. The SRGs inscribed on the azobenzene‐PDMS bilayer were able to guide focal adhesion alignment and orientation of epithelial cells. Erasure of the SRG topography in the presence of live cells enabled the investigation of the effect of topographical change on epithelial cells. The PDMS coating improved the uniformity of the surface topography upon erasure with live cells. The topography erasure was not as efficient in cell culture environment as it was in dry environment. Yet the surface pattern could be modulated during the cell growth, which enabled reorienting the focal adhesions, paving way to dynamic azobenzene‐based cell culture platforms. The coating also enabled further functionalization of the platform with proteins, offering a route toward independent control of topographical and biochemical signals. These results are a step closer to a dynamically controlled photosensitive cell culture platform, which is envisioned for in vitro studies of cell–material and cell–cell interactions.

## 2 Results and Discussion

### 2.1 Characterization of SRG Inscription and Erasure on DR1g‐PDMS Bilayer Structures

To assemble the platform, glass coverslips were first coated with photopatternable amorphous thin layer of Disperse Red 1‐containing molecular glass^[^ ^30^ ^]^ (DR1g; thickness 480 ± 20 nm) that were further coated with PDMS. The resulting DR1g‐PDMS bilayer structure was used as the light‐responsive cell culturing platform (Figure 1b) where DR1g functions as the light‐responsive part, and we hypothesized the PDMS coating to improve the material properties in cell culturing environment, enabling also protein pattering of the bilayer without hindering the photoresponse.

To study the effect of the PDMS (base:curing agent ratio 10:1) on the SRGs formation, three different PDMS pre‐polymer dilutions in *n*‐hexane (0.02, 1, and 50 wt%) were tested with the same spin coating parameters. These samples are denoted here as DR1g‐PDMS_*x*_, where *x* stands for the PDMS concentration in hexane. The thickness of the PDMS layer was measured by ellipsometry and profilometry. Ellipsometry was used for accurate measurement for the 0.02 and 1 wt% layers, and for thickest PDMS layer (50 wt%), profilometer was used. The thicknesses were ≈4.5 μm (DR1g‐PDMS_50_), ≈65 nm (DR1g‐PDMS_1_), and ≈20 nm (DR1g‐PDMS_0.02_). The DR1g‐PDMS bilayer was photopatterned using light interference lithography in the Lloyd's mirror configuration,^[^ ^8^, ^31^, ^32^ ^]^ which induces mass migration in DR1g and surface deformation of the PDMS coating to form the SRGs (Figure 1b). The periodicity of the interference pattern is determined by the wavelength and the angle between the mirror and the laser beam. By varying the angle, SRGs with different periodicities can be achieved (roughly in the range 300 nm–10 μm). Here, the periodicity was set to 1 μm, as this periodicity has been previously used in controlling the alignment of epithelial cells for the same material.^[^ ^16^ ^]^ In situ monitoring of the SRG formation within the different DR1g‐PDMS bilayers was conducted via diffraction efficiency (DE) measurements. The thickness of the DR1g film (480 ± 20 nm) was chosen to be large enough so that the SRG formation was independent from small variations in the layer thickness (Figure S1, Supporting Information).^[^ ^33^, ^34^ ^]^ Thus, the differences in DE were solely due to differences in the PDMS layer. The samples were imaged with atomic force microscopy (AFM), to confirm the SRG formation.

The DE curves during SRG inscription on the DR1g‐PDMS bilayers are shown in Figure 1c. From these images, it can be observed that the DE systematically decreases with increasing PDMS layer thickness. AFM imaging confirmed the formation of SRGs for DR1g‐PDMS_0.02_ and DR1g‐PDMS_1_ with the expected periodicity of 1 μm (Figure 1d,e). Surface modulation depth was over 400 nm for DR1g‐PDMS_0.02_, while for DR1g‐PDMS_1_, the modulation depth was significantly decreased and reached 160 nm. With DR1g‐PDMS_50_, SRGs were not formed onto the outer surface of the PDMS coating since no sinusoidal pattern could be observed with AFM. The 8% DE can be attributed to the grating formation at the DR1g/PDMS interface.^[^ ^35^ ^]^ In agreement with previous reports, when the azobenzene‐containing film is between the glass substrate and a protective coating, it has stronger constraints to move efficiently.^[^ ^36^ ^]^ The SRG formation requires material mass migration, thus we believe that the presence of thick PDMS layer resulted in increased hindrance to the complex stress field DR1g is subjected to during SRG formation. Based on these results, we can conclude that the PDMS coating with thickness below 100 nm on top of a thin DR1g film does not inhibit the formation of SRGs but alters the dynamics of its formation.

**Figure 2:**
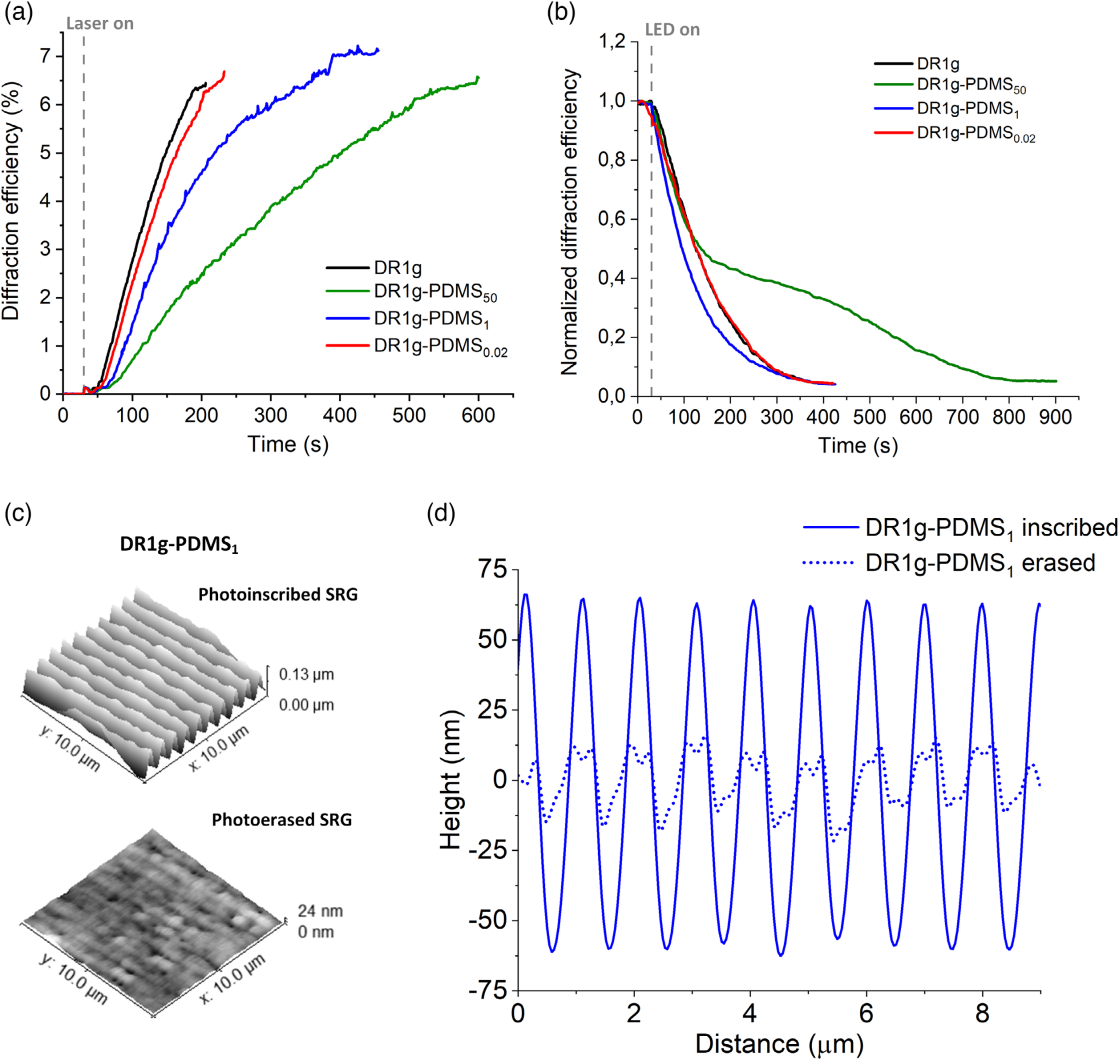
Diffraction efficiency curves for different thicknesses of PDMS layer during a) SRG inscription with intensity of 500 mWcm^−2^ and b) SRG erasure with a 530 nm LED. c) AFM images of the surface topography of DR1g‐PDMS_1_ after inscription (top) and erasure (bottom). d) Cross‐sectional profiles of the SRG modulation depth for photoinscribed (solid line) and erased (dashed line) DR1g‐PDMS_1_.

After the SRGs are formed, the topography is stable for at least a year at temperatures below the glass‐transition temperature of DR1g (71 °C) but can be erased thermally or with a uniform light beam with wavelength matching the absorption band of DR1g.^[^ ^11^, ^30^, ^37^, ^38^ ^]^ As direct heating cannot be localized and is not compatible with cell culture conditions, we chose to erase the SRGs with visible light (530 nm LED). To study the dynamics of the SRG erasure, we monitored the DE during the erasure for samples exhibiting the same initial DE value (≈7%, **Figure** **2**a). The erasure dynamics are shown in Figure 2b. All samples reached similar (≈0.5%) DE value at the end of the process, hence the PDMS layer thickness does not seem to affect the effectiveness of the erasure process in terms of DE values. However, clear differences in erasure dynamics can be observed between the different PDMS thicknesses. Interestingly, the DE decreased systematically faster for DR1g‐PDMS_1_ compared to other samples, suggesting that the PDMS layer with proper thickness can even accelerate the topography erasure. With DR1g‐PDMS_50_ the erasure dynamics differed from other samples and the DE decreased relatively slowly, in a two‐step process. We may speculate that the second part of the process is dominated by the interaction with the thick PDMS layer. Similarly to the inscription process, we believe that this behavior is due to the increased surface constraint, which slows down the material reorganization at the interface.

The samples were imaged with AFM, which confirmed that the topography was reduced up to 85% of the initial value for DR1g‐PDMS_1_ as Figure 2c shows. The surface profiles in Figure 2d further highlight the difference between erased and nonerased topographies. The decrease in modulation depth was ≈70% for DR1g‐PDMS_0.02_ (Figure S2, Supporting Information). Even if the DE‐values reached a similar value, AFM shows that with DR1g‐PDMS_1_, the erased topography reached a lower modulation depth. This served as the motivation to select DR1g‐PDMS_1_ for cell culture studies. It has been previously observed by Lagugné‐Labarthet et al. that with deeper gratings, the erasure with uniform laser beam is less effective in comparison to lower SRGs.^[^ ^39^ ^]^ Thus, the complete erasure of the topography might be more difficult achieve for DR1g‐PDMS_0.02_ surface, as determined from the higher remnant gratings observed on this sample. With DR1g‐PDMS_1_, the grating depth favored a fast erasure process, leading to a lower residual topography, limiting the irradiation time needed during cell‐growth experiments. Based on previous reports,^[^ ^40^ ^]^ the gratings were also deep enough to induce cell response in terms of alignment. We also speculate that the thicker PDMS layer acts as a better protective layer to separate the DR1g layer from the cells. Therefore, DR1g‐PDMS_1_ was chosen for the experiments that follow.

### 2.2 SRG‐Guided Cell Alignment

Cells sense physical properties of their environment and mechanical forces at their surface, but these forces are transduced also deeper into the cells, even to the nucleus.^[^ ^41^ ^]^ The main sites for sensing are cell–ECM contacts, mainly focal adhesions, which are multiprotein complexes at the cell membrane. The formation of focal adhesions at the cell–ECM interface regulates the cell attachment, alignment and migration.^[^ ^40^ ^]^ A calcium‐dependent transmembrane protein, E‐cadherin, is one of the molecules found at the cell–cell contact sites.^[^ ^42^, ^43^ ^]^ E‐cadherin is present especially in adherens junctions and plays a key role at the cell–cell interface during formation of tight and polarized epithelium.^[^ ^44^ ^]^

**Figure 3:**
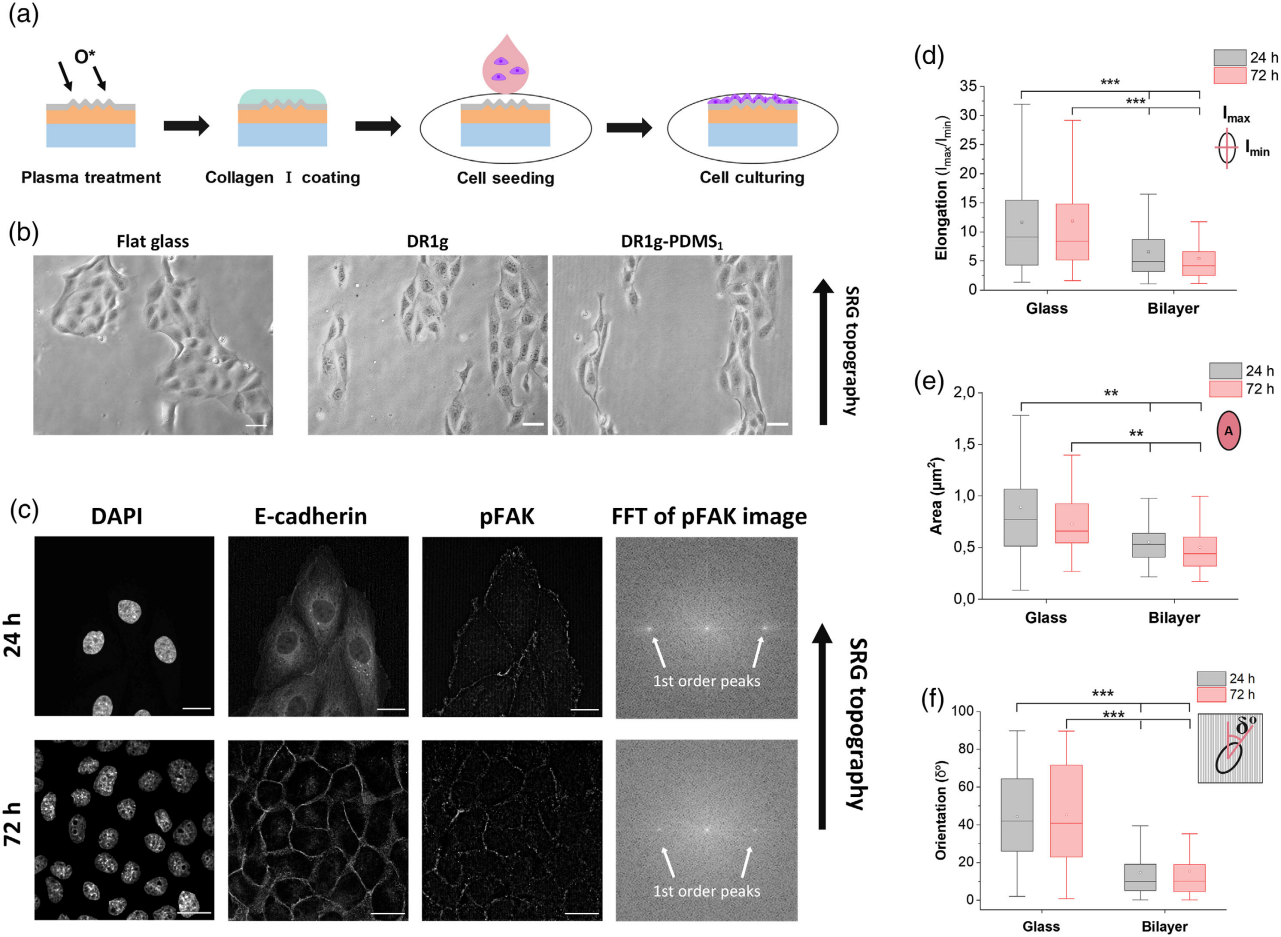
a) Schematic representation of DR1g‐PDMS_1_ sample preparation for cell culture experiments. b) Optical microscopy images of MDCK II cells on a flat glass substrate and surface‐patterned films of DR1g and DR1g‐PDMS_1_ after 24 h from cell seeding. Black arrow indicates the SRG topography direction. Scale bars: 50 μm. c) Immunolabeled MDCK II cells on surface‐patterned DR1g‐PDMS_1_ bilayer at different time points (24 h, 72 h). The labels used were DAPI (chromatin), E‐cadherin (cell–cell junctions) and pFAK (mature focal adhesions). The organization of focal adhesions was analyzed with fast Fourier transform (FFT) of the pFAK image showing the periodicity in the image (indicated by 1st order peaks). Black arrow indicates the SRG topography direction. Scale bars: 20 μm. Focal adhesion d) elongation, e) size, and f) orientation, showing maximum, mean and minimum values on a flat glass coverslip and surface patterned DR1g‐PDMS_1_ after 24 and 72 h cell culturing.

To study whether the microtopography on DR1g‐PDMS_1_ bilayer could guide collective cell alignment, Madin–Darby canine kidney type II (MDCK II) epithelial cells were seeded on the SRG and their alignment to the underneath microtopography was studied. This cell line provides a good model for studying cellular collective behavior.^[^ ^45^ ^]^ While the mechanotransduction of single cells on microtopographies has been largely investigated,^[^ ^46^, ^47^, ^48^ ^]^ such behavior has not yet been fully characterized for cell collectives, for which concerted movements happen without complete disruption of their cell–cell contacts. **Figure** **3**a shows the schematic representation of the sample preparation. Briefly, the DR1g and PDMS were subsequently spin‐coated to form the bilayer structure and the SRGs were inscribed as previously described. After patterning, the surface was made hydrophilic with an oxygen plasma treatment to improve protein attachment to the surface. The surface was then coated with collagen I to improve cell adhesion onto the surface^[^ ^16^ ^]^ and MDCK II cells were seeded on the samples and cultured up to 72 h. The cell migration along the microtopography was followed by time lapse microscopy. Cells aligned along the microtopography already during the first 24 h post seeding on DR1‐PDMS_1_ (Supporting video 1). After 24 h, the cells were forming small colonies, elongating along the pattern direction on both bare DR1g and DR1g‐PDMS_1_, indicating that the PDMS layer does not inhibit cells from sensing the underneath topography (Figure 3b). The cells formed a confluent cell monolayer at 72 h time point (Figure S3, Supporting Information).

The cellular response to the microtopography in terms of cell–material and cell–cell interactions was further investigated by immunolabeling the MDCK II cells at different time points. The nuclei were stained with DAPI for distinguishing single cells. The cell–cell interaction was studied by detecting intracellular localization of E‐cadherin. After 24 h from cell seeding, the cell nuclei were round, but the cells had an elongated morphology along the surface microtopography as observed from E‐cadherin localization (Figure 3c). In addition, E‐cadherin accumulated in the cytoplasm, thus the cells were not yet forming mature cell–cell junctions.^[^ ^49^ ^]^ At 72 h timepoint, the morphology of the cells was less elongated than at the 24 h timepoint. As expected, after 72 h, cells were forming a uniform cell layer and E‐cadherin localized to the cell–cell interface, showing strong cell–cell interactions on the bilayer surface. The loss of E‐cadherin, instead, indicates epithelial‐mesenchymal transition (EMT), where epithelial cells lose their phenotypic characteristics and transform to nonpolarized mesenchymal cells, which are more motile and potentially invasive.^[^ ^50^ ^]^ Since E‐cadherin was localized at the cell–cell interface, cells on the bilayer surface were forming a tight epithelium layer after 72 h.

Focal adhesion kinase (FAK) is one of the first molecules present in focal adhesion development and its phosphorylation indicates the formation of mature focal adhesions.^[^ ^41^ ^]^ Thus, morphological parameters of focal adhesions were studied by immunolabeling phosphorylated FAK (pFAK). Focal adhesions were observed at the cells edge in the basal plane after 24 h from seeding and their distribution was further analyzed by using fast Fourier transform (FFT). FFT converts the spatial image information into frequency space, where periodic features are emphasized yielding a specific pattern of frequencies.^[^ ^51^ ^]^ The analysis showed that first‐order frequency peaks can be detected after 24 h from cell seeding (Figure 3c, FFT of pFAK image) showing periodic distribution of the image features (pFAK) in accordance to our previous studies.^[^ ^16^ ^]^ After 72 h, pFAK was still observed at the cell edges, though after forming uniform cell layer, cell movements are more restricted. In the FFT for the focal adhesion channel the first‐order frequency peaks were still visible, indicating that focal adhesions were periodically distributed, and cells were still perceiving the information from the topographical cue.

Next, we analyzed the elongation, size, and orientation of the focal adhesions (Figure 3d–f). The elongation was determined as the ratio of the maximum and minimum moments of inertia of the focal adhesion equivalent ellipse (*I*_max_/*I*_min_) and values above 1 indicated elongated focal adhesions. Cells cultured on both glass substrate and DR1g‐PDMS_1_ with SRGs had elongated focal adhesions after 24 h of cell culturing and they were elongated still after 72 h (Figure 3d). However, when cultured on the microtopography, the focal adhesions had rounder morphology and smaller size compared to the glass coverslip (Figure 3d,e). We also analyzed the orientation of focal adhesions in the direction of the microtopography (Figure 3f). The orientation was defined as the angle between the surface pattern direction (vertical axis for flat substrate) and the maximum axis of inertia (δ°) of focal adhesion. For cells oriented along the SRG topography this angle was set to 0°. As expected, focal adhesions on a flat glass coverslip had random orientation and the mean value for the *δ*° was 45° with broad distribution on both 24 and 72 h timepoints. Cells adhering on microtopography showed oriented focal adhesions along the surface topography already 24 h post seeding. The focal adhesions were still oriented along the underneath microtopography after 72 h from cell seeding and the mean *δ*° value was 15° at both timepoints.

Photoresponsive azobenzene‐based materials have been used previously with other cell lines, such as NIH‐3T3 fibroblasts,^[^ ^14^, ^52^, ^53^ ^]^ HUVEC endothelial cells,^[^ ^54^ ^]^ neurons,^[^ ^17^, ^55^ ^]^ and mesenchymal stem cells.^[^ ^56^, ^57^ ^]^ These studies show that single cells can respond to the SRG topography and guide the cell alignment. The results shown here demonstrate that the addition of the PDMS coating in the light‐responsive bilayer did not impair collective cellular mechanosensing of the microtopography, and the cells presented focal adhesions aligned to the direction of the microtopography. The cells cultured on a flat glass coverslip had bigger and more elongated focal adhesions, however they were randomly oriented. The smaller focal adhesions on patterned bilayer indicate that the cells have weaker adhesion on the bilayer than on glass.^[^ ^58^ ^]^ Since glass coverslips are more hydrophilic than PDMS, cells have stronger attachment on glass, despite the increased hydrophilicity of the bilayer films after oxygen plasma treatment. In addition, the focal adhesion size is limited by the actual area of the cell membrane in contact with the surface, which defines the maximal area available for their formation. With sinusoidal SRGs, this area is confined to the surface on the top of the gratings, which can limit focal adhesion size.^[^ ^59^ ^]^ The weaker cell–material interaction can also affect the elongation of focal adhesions on the bilayer surface, which is why on the bilayer focal adhesions were not as elongated as on the glass. Despite the weaker cell–material interaction on the patterned bilayer, the focal adhesions were orienting along the microtopography, thus cells were responding to the underneath SRG topography. These results show that the SRG topography on the bilayer structure can guide epithelial cell alignment collectively and promote the formation of the epithelium.

### 2.3 Erasure of SRG Topography with Live Cells

We next sought to erase the SRG microtopography in the presence of live cells to investigate the cell responses to a dynamic topographic change. The influence of dynamic topographic signals on collective cell behavior has not yet been broadly investigated. In this case, instead of using the LED, the microtopography was erased with a fluorescent lamp of a confocal microscope (filtered in the blue region of the visible spectrum), which enables the observation of live cells right after the measurement. This setup was deemed practical for biological environment since most microscopes can be equipped with environment control, suitable for live cell culture.^[^ ^14^ ^]^

**Figure 4:**
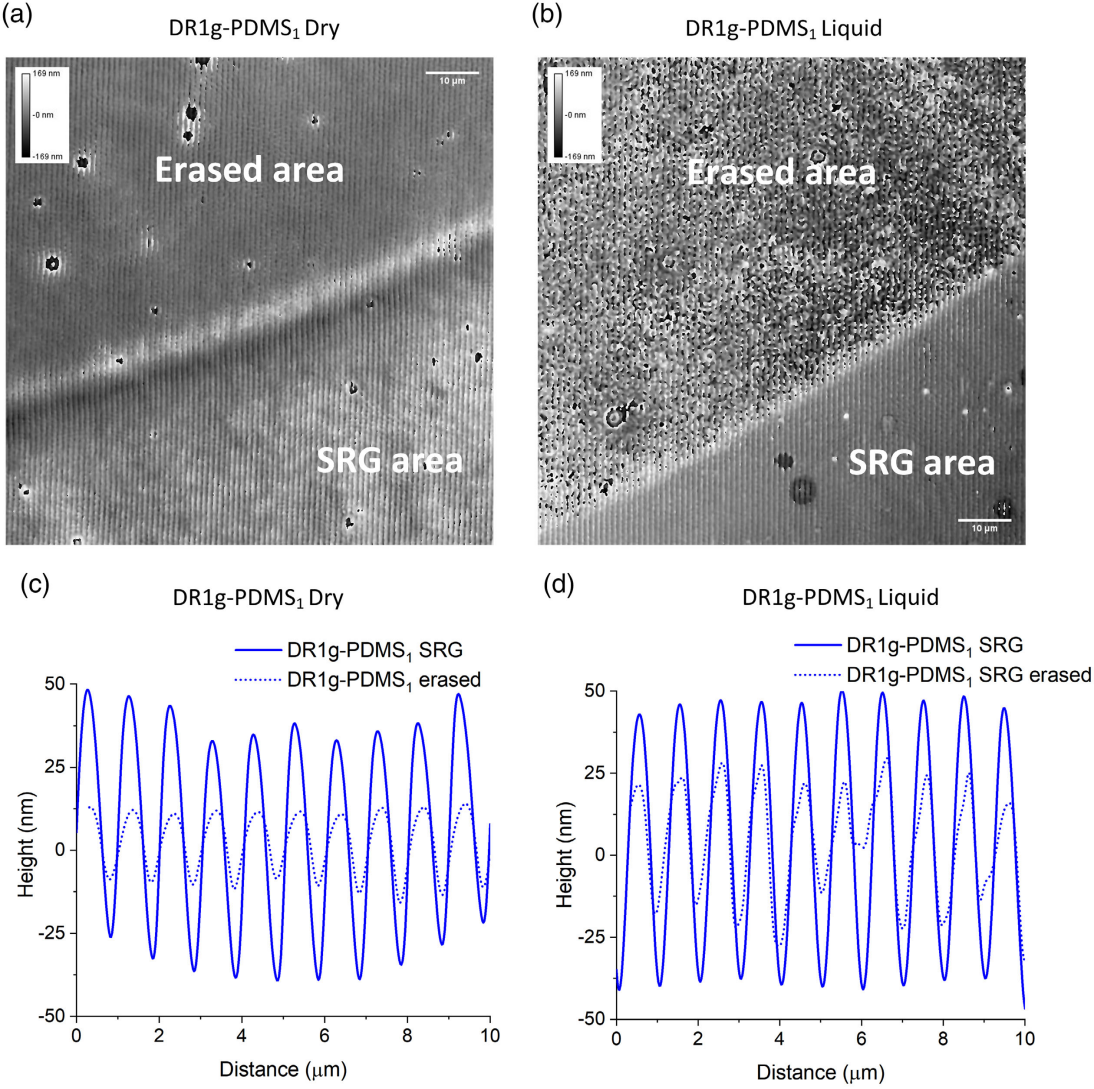
DHM images of SRG topography on DR1g‐PDMS_1_ after erasure with a fluorescent lamp of a confocal microscope filtered in the blue region (470 ± 40 nm) a) in dry environment and b) in liquid environment. Irradiation time: 5 min. Scale bars: 10 μm. Surface profiles of the SRG topography on DR1g‐PDMS_1_ after erasure c) in dry environment and d) in liquid environment.

The erasure was first conducted in dry and liquid environments at room temperature without cells, to set the erasure parameters. Illumination with the fluorescent lamp clearly resulted in a distinguishable circular area in both dry and aqueous environment, as seen from bright field images (Figure S4, Supporting Information) and digital holographic microscopy (DHM) images, which yield quantitative results about the surface profile (**Figure** **4**a,b).^[^ ^60^ ^]^ DHM was used to monitor the surface, allowing a fast and quantitative characterization of the surface topography over a bigger area compared to AFM. DHM images showed reproducible decrease in modulation depth within 5 min of irradiation. In dry conditions, the modulation depth decreased ≈75% from the initial value (Figure 4c), indicated by surface roughness decrease from 56 nm to 14 nm. In liquid environment the modulation depth of the erased area decreased by ≈50% (Figure 4d), and in addition, the (partially) erased surface was significantly rougher and exhibited round surface features (Figure S5, Supporting Information). We speculate that this phenomenon might be due to interactions between water molecules and the material surface as also hypothesized by Rianna et al.^[^ ^14^ ^]^ and Rocha et al.^[^ ^61^ ^]^ However, the phenomenon behind the formation of the round structures needs to be studied further before conclusive statements can be made.

**Figure 5:**
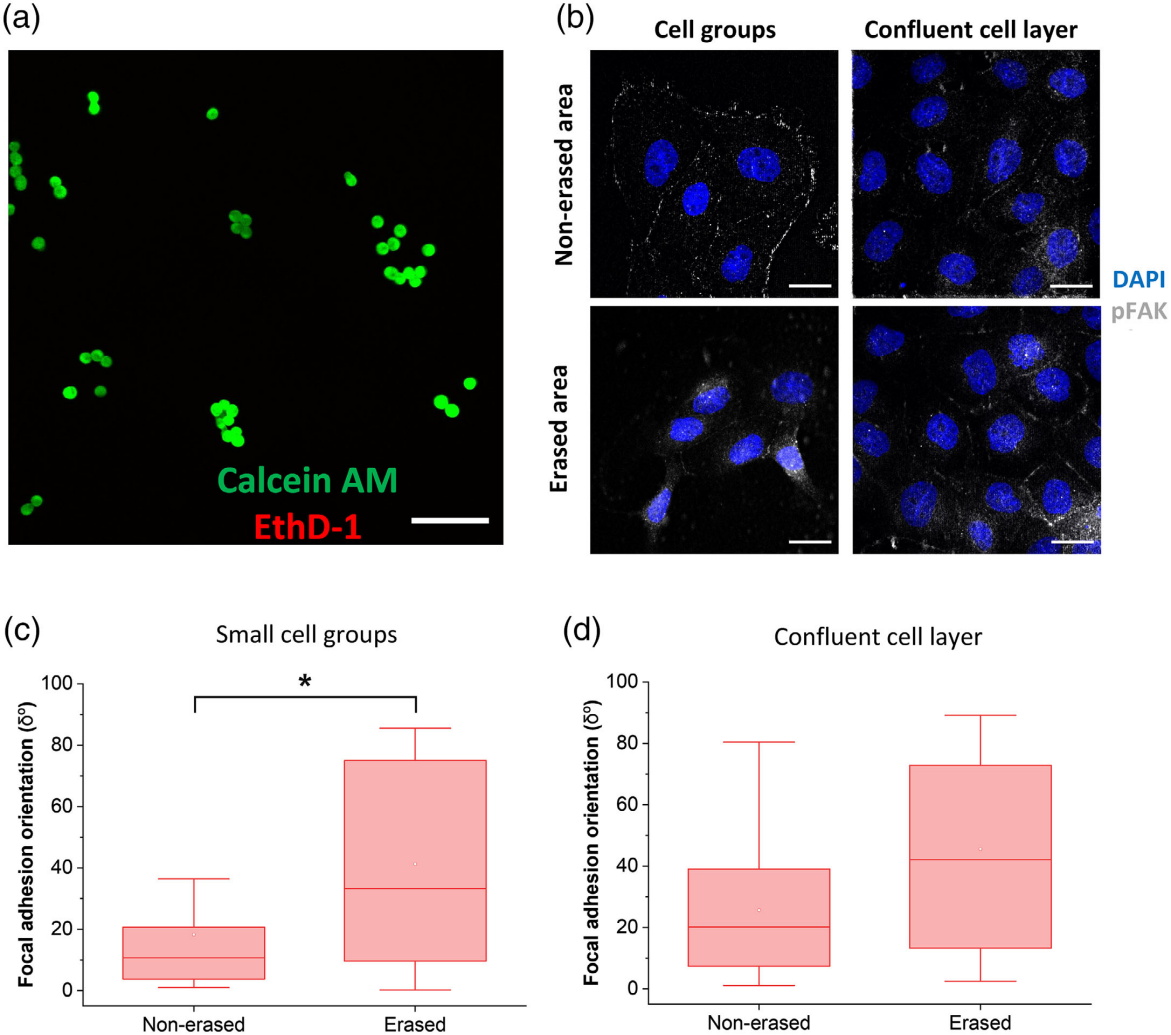
a) Live/Dead viability assay image after topography erasure. Live cells are indicated as green fluorescent signal (calcein AM) and dead cells as red fluorescent signal (EthD‐1). Scale bar: 100 μm. b) Immunolabeled MDCK II cells on DR1g‐PDMS_1_ bilayer samples on nonerased (top) and erased (bottom) SRG topography in the presence of small cell groups (left) and confluent cell layer (right). Erasure was conducted by illuminating the samples with a fluorescent lamp of a confocal microscope filtered in the blue region (470 ± 40 nm) for 5 min. Labels used were chromatin (DAPI stain, blue), and pFAK (grey). Scale bars: 20 μm. Focal adhesion orientation when topography was erased in the presence of c) small cell groups and d) confluent cell layer.

Next, the topography was erased in the presence of living cells. To this end, MDCK II cells were seeded on the SRG topography and cultured for 24 h prior to the erasure, to allow cell orientation along the microtopography. The samples were illuminated with the fluorescent lamp of a confocal microscope with medium on top at 37 °C in a humidified atmosphere for 5 min, fixed after 2 h from erasure and immunolabeled. The partial photoerasure was confirmed by DHM after cell removal by trypsin treatment (Figure S6a, Supporting Information). In the presence of the PDMS layer, the erasure was more uniform in comparison to the bare DR1g, yielding significantly lower number of the round surface features described above (Figure S6b, Supporting Information). The possible phototoxicity on cells was also studied. For this experiment, cells were seeded on samples in which DR1g was spin‐coated to the bottom side of the glass coverslip and glass substrate was at cell–material interface. Such control sample ensured that a similar light intensity reached the plane of the cells as in the erasure process, but no topographical change could be produced at the cell adhesion sites. The control samples were illuminated with the fluorescent lamp for 5 min, and after 3 h form erasure Live/Dead viability/cytotoxicity assay was performed (**Figure** **5**a). This showed that no major acute phototoxic effects on cell viability could be observed as no dead cells (red) could be seen in the erased areas similarly to the nonerased areas (Figure S7a, Supporting Information). When studying phototoxicity effects on cell morphology, PDMS was spin‐coated on the other side on top of the control sample, at the cell–material interface, to ensure similar adhesion properties. No significant difference in cell morphology could be observed within 2 h from irradiation (Figure S7b, Supporting Information).

Figure 5b shows the cell morphology after erasure for smaller cell groups (left) and more confluent cell layers (right). The cell groups had a less spread morphology and smaller size after erasure, which might indicate partial loss of substrate attachment after the topography changes.^[^ ^62^ ^]^ In addition, pFAK was observed to be more concentrated in the cell center rather than in cell edges after the erasure. When microtopography was erased underneath a uniform epithelial cell layer, no significant morphology change could be observed. This observation suggests that, when strong cell–cell connections (indicated by E‐cadherin in Figure S8a, Supporting Information) are formed, epithelial cells in a monolayer do not immediately rearrange as a response to loss of guiding surface topography, at least within 2 h posterasure. Quantification of the focal adhesion orientation was conducted similarly as reported above (Section 2.2). The orientation data showed that focal adhesions were more randomly oriented after erasure with small cell groups (Figure 5c). However, no differences could be observed in the case of confluent cell layer (Figure 5d). This result indicates that smaller cell groups can sense the light‐induced topographical change and re‐orient the focal adhesions accordingly.^[^ ^14^ ^]^ Erasure seemed to have no effect on elongation and area of the focal adhesions (Figure S8b,c, Supporting Information).

Even if the topography erasure was only partial with the lamp of the confocal microscope in the presence of liquid, the microtopography and the roughness of the surface could be changed. The topography change affected the morphology and focal adhesion orientation with small cell groups. However, no collective morphological response or focal adhesion orientation could be observed at least during the time span of 2 h. The cells remained attached and viable on the erased surface after irradiation, which indicates that the erasure process does not cause cell death within this timespan. The possibility to erase surface topography with live single cells has been previously studied by Rianna et al.^[^ ^14^ ^]^ The authors also observed the formation of round‐shaped surface structures on the photosensitive layer (poly(DR1‐methacrylate)), which they attributed to light scattering effects and the interactions between water molecules and the polymer layer. In their case, NIH‐3T3 fibroblasts cells used in their study were motile within 45 min after irradiation. Overall, the examples of using azobenzene‐based materials for cell culture applications still remain scarce, yet we foresee them to become more common in the near future. The presence of the PDMS protective layer seems to improve the erasure process and limit the formation of unwanted surface features, in addition to potentially protecting the cells from possible harmful materials, even if further studies are needed to understand the full potential of the reported bilayer structures.

### 2.4 Cell Alignment Guided by Collagen Micropatterns

**Figure 6:**
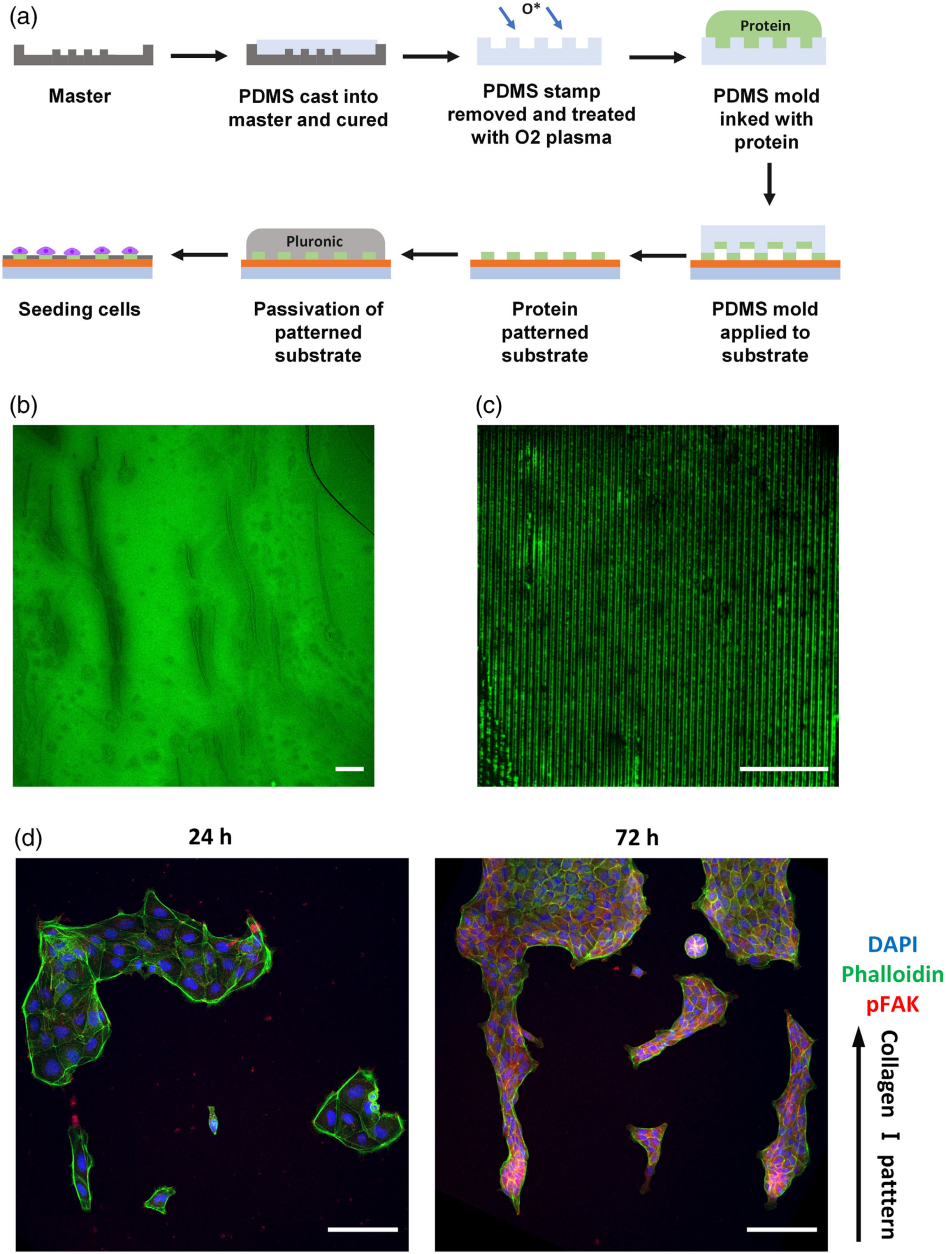
a) Representation of microcontact printing and cell seeding. FITC‐conjugated gelatin patterns with b) flat stamp and c) patterned stamp on DR1g‐PDMS_1_ bilayer. d) Immunolabeled MDCK II cells on microcontact printed DR1g‐PDMS_1_ bilayer samples at different time points (24 h, 72 h). Labels used were chromatin (DAPI stain, blue), actin (phalloidin stain, green) and pFAK (red). Black arrow shows the direction of collagen I pattern. Scale bars: 50 μm.

Next, we wanted to demonstrate the ability to pattern the bilayer also with protein patterns, enabling independent control of the biochemical and topographical cues. This would allow the comparison of different patterning techniques and would also improve the functionalization of the cell culture platform. The PDMS layer on DR1g enables the combination of different patterning techniques. To compare the guidance effect of the microtopography and ECM protein patterns, microcontact printing (μCP) was used first to print protein patterns on a flat DR1g‐PDMS_1_ surface.^[^ ^63^ ^]^ Stamps with flat surface (flat stamp) and stamps with micropatterns (patterned stamp) were used. **Figure** **6**a shows graphical representation of the printing process. To tune and evaluate the quality of the printing process, fluorescein (FITC)‐conjugated gelatin was used for protein patterns on DR1g‐PDMS_1_ bilayer. After μCP, the bilayer surface was imaged with a confocal microscope and images of the resulting gelatin patterns with flat and patterned stamps are shown in Figure 6b,c. With flat stamps, visible areas with gelatin were achieved, even though wrinkles on the PDMS surface could be observed (Figure 6b), most likely due to the oxygen plasma‐induced highly oxidized SiO_2_ layer on top of the PDMS.^[^ ^64^ ^]^ When using patterned stamps, parallel stripes (≈2 μm wide) of fluorescent gelatin were observed (Figure 6c).

Once the μCP parameters were set, cells were seeded on collagen I ‐patterned DR1g‐PDMS_1_ bilayer. Prior to cell seeding, the samples were passivated with pluronic F‐127 (PF‐127), to avoid unwanted attachment of the cells to nonpatterned areas (Figure 6a). The cells were cultured on the samples with protein patterns for 24 and 72 h and immunostained at these timepoints. After 72 h from cell seeding, only few cells could be observed on the passivated sample surface, thus the passivation was effective. With collagen I‐coated sample, cells adhered well on the surface and formed uniform cell layer at 72 h time point (Figure S9a, Supporting Information). With flat stamps the cells adhered only to the stamped areas and the boundary between stamped and passivated area was clear already 24 h after cell seeding (Figure S9a, Supporting Information). After 72 h, however, the cells did not form a confluent cell layer on the whole collagen stamped area, which could be due to uneven patterning of collagen I.

For areas with parallel collagen I stripes, single cells were adhering along the collagen pattern, however, cell groups appeared to have a random orientation (Figure 6d). After 72 h, the cells were growing along the collagen pattern, but they were also attaching to passivated surface outside the protein stripes. It has been shown that epithelial cells can form cell bridges over nonadhesive areas during collective cell migration, which explains the spreading of epithelial cell layer also on the passivated areas.^[^ ^65^ ^]^ These epithelial bridges are under a notable amount of tension, which shows as activation of actin network. This can be seen as accumulation of actin stress fibers along the cell edges, potentially indicating increased tension in these areas. Image quantification supported these observations, as the focal adhesion orientation seemed to be randomly distributed on the protein pattern (Figure S9b, Supporting Information). Thus, the collagen I pattern does not orient the focal adhesions similarly as the microtopography does. Instead of orienting the focal adhesions, the protein pattern specifies the areas where the cells attach to and in this way guides the cell migration and growth. It has been previously reported that ECM protein pattern can organize focal adhesions in the direction of protein alignment.^[^ ^66^ ^]^ Interestingly, no such orientation can be observed based on our results. This can be due to the width of the protein pattern used. With stripes that are wider than mature focal adhesions (≈2 μm), it has been observed that the focal adhesions are more elongated and oriented along the lines.^[^ ^67^ ^]^ Thus with this length scale, we speculate that the cell orientation rises from minimized cell adhesion on passivated regions between protein patterns as reported by Buskermolen et al.^[^ ^67^ ^]^ and not the focal adhesion alignment.

**Figure 7:**
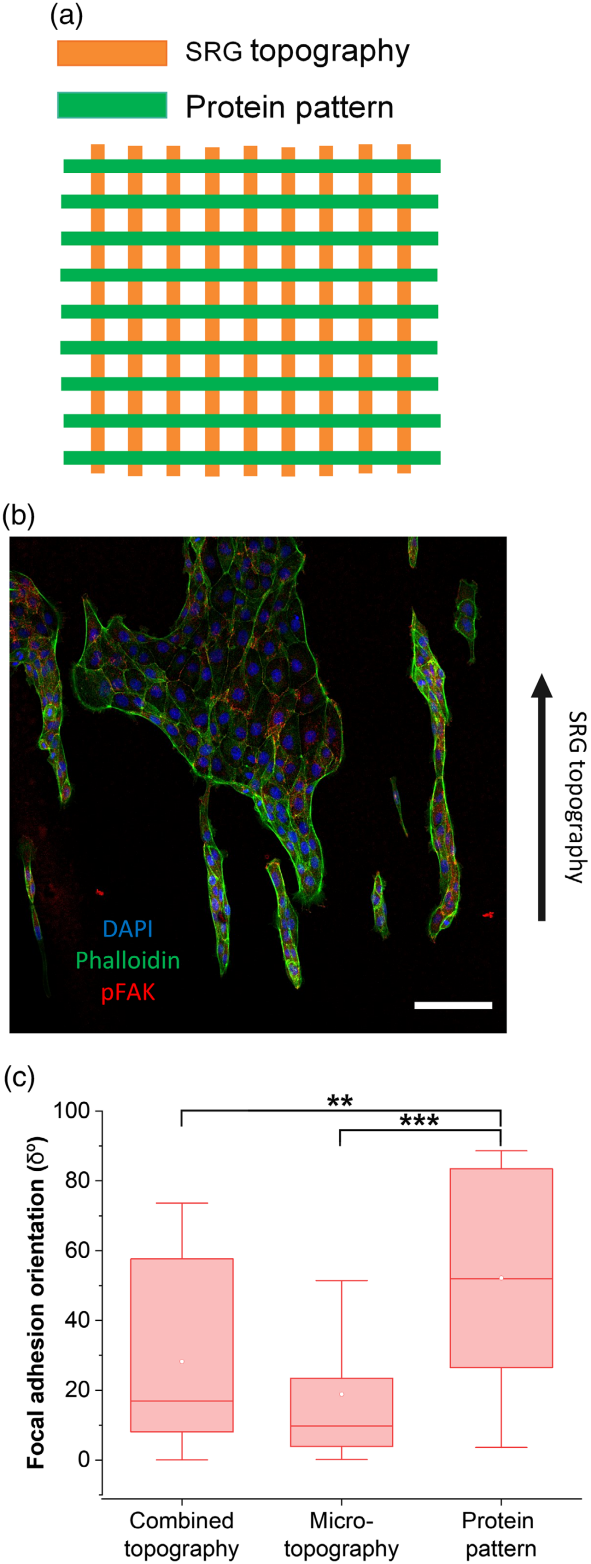
a) Graphical representation of combined microtopography and protein pattern. b) Immunolabeled MDCK II cells on DR1g‐PDMS_1_ bilayer with combined topography. Labels used were chromatin (DAPI stain, blue), actin (phalloidin stain, green) and pFAK (red). Black arrow shows the direction of the SRG topography. Scale bar: 50 μm. c) Focal adhesion orientation on combined topography, microtopography, and protein pattern.

To further study the differences between the microtopography and protein patterns, MDCK II cells were seeded also on a combined topography, where collagen I protein pattern was perpendicular to SRG microtopography (**Figure** **7**a). Already after 24 h from cell seeding it was evident that cells were aligning along the microtopography rather than protein pattern as observed from Figure 7b. The focal adhesion orientation was also quantified with respect to the SRG topography (*δ*° for microtopography is 0° and for protein pattern 90°). Image quantification results in Figure 7c showed that the focal adhesions were randomly oriented on combined topography and the focal adhesions seemed to have acquired orientation that was between the microtopography and the protein pattern. However no significant difference could be observed between the combined topography and microtopography. The focal adhesion orientation on protein patterned substrate differed significantly compared to both combined topography and microtopography. When comparing the median *δ*° values, we found out that for combined topography (≈17°) this value was closer to microtopography value (≈10°) than protein pattern value (≈52°), indicating that the microtopography is dominating over the protein pattern. Charest et al. have also observed that the mechanical surface topography preferentially drives cell alignment over protein pattern.^[^ ^26^ ^]^ Since the protein pattern is dependent on the success of the microcontact printing process, the pattern can have defects creating discontinuous protein areas. The SRG microtopography is well defined, thus cells orient along the sinusoidally modulated surface rather than the protein pattern. In addition, the size‐scale of guiding features is different since in SRG the ridge‐to‐ridge distance of the features is 1 μm, but protein patterns have a width of 2 μm. And as stated above, the protein patterns in this length scale are not able to guide focal adhesion orientation, whereas the 1 μm microtopography can induce focal adhesion alignment.

This result shows that cells could be seeded on a specified area on the platform with the collagen I printing, since cells were only attaching to the protein patterned areas and not to the passivated areas (Figure S10, Supporting Information). μCP was able to create protein patterns that can guide epithelial cell groups’ attachment along the protein patterns, but not the focal adhesion orientation. The underlaying mechanism for cell alignment on protein pattern differed from the one in microtopography guided alignment. In addition, on combined topography, the microtopography guided orientation was stronger than the protein patterns. Further optimization of the protein printing and passivation is needed to fully exploit its potential. With wider protein patterns the cell alignment could be more evident, which could also affect the cell orientation on the combined topography. Protein patterning would enable the functionalization of the platform for different applications and allow control of topographical and biochemical cues independently.

## 3 Conclusions

The platform presented here consists of a light‐responsive DR1g film and a thin PDMS coating, which allowed independent control of the light responsivity and the stability of the material in cell culture environment. Together these layers formed a bilayer structure, which allowed surface topography modification with light‐induced movements of azobenzene‐containing DR1g film. The SRG topography was efficiently photoinscribed and ‐erased in the presence of 65 nm thick PDMS layer (DR1g‐PDMS_1_). When MDCK II epithelial cells were seeded on photopatterned DR1g‐PDMS_1_, the SRG topography could guide focal adhesion orientation along the surface topography still after formation of uniform epithelial layer. The surface topography could be altered in the presence of live cells with a fluorescent lamp of a confocal microscope, enabling noninvasive control over the surface topography. Despite the SRG topography erasure was only partial, the topography could still be changed without causing cell detachment or cell death. Thus, light‐mediated erasure is a promising strategy to control the material topography dynamically for real‐time cell experiments, which can be conducted with conventional microscopy setups. The platform could be further patterned with proteins, enabling individual control of the topographical and biochemical cues and further functionalization for different applications. We believe that in the future, this platform can be used to understand the complex cell–material interactions and cell behavior in dynamic environments, taking a step toward the design smart stimuli‐responsive materials for dynamic control of the biomaterial properties.

## 4 Experimental Section

4.1

4.1.1

##### Sample Preparation

A bilayer of azobenzene‐containing Disperse Red 1 molecular glass (DR1g, Solaris Chem Inc.) and polydimethylsiloxane (PDMS, SYLGARD 184, Dow) was prepared on square glass coverslips by spin coating (Laurell Technologies Corporation). The glass coverslip was first ultrasonicated twice in acetone for 10 min. Solution of DR1g with concentration of 9% (w/v) in chloroform was prepared. The solution (35 μl) was deposited on the glass coverslip (22 × 22 mm^2^) at 1500 rpm for 30 s. PDMS was prepared by mixing prepolymer silicone elastomer base and curing agent in 10:1 ratio. The uncured PDMS was diluted in n‐hexane to create 50, 1, and 0.02 wt% solutions. The solutions were dispensed over the thin DR1g film at 6000 rpm for 150 s and cured at 55 °C for 1.5 h. Samples for thickness measurement were first prepared by spin coating PDMS solution on a silicon substrate as described above. Thickness of the produced PDMS film was measured with reflection ellipsometry (J.A, Woollam VASE). The 50 wt% PDMS solution formed too thick film for ellipsometry measurement, thus the thickness was measured with Stylus profilometry (Veeco Dektak 150+). For both techniques the resolution limit is in sub‐nanometer range.

##### Surface Relief Grating Inscription and Erasure

The bilayer structures were photopatterned with interference lithography in Lloyd's mirror configuration. Inscription of surface relief gratings (SRGs) was done using a 488 nm continuous‐wave laser (Coherent Genesis CX488‐2000) with circular polarization and an intensity of 300 mWcm^−2^ over an area of 0.50 cm^2^. The microtopography period Λ was set to 1 μm and it was determined by Λ = *λ*/2sin*ϑ*, where *λ* is the wavelength of the laser and *ϑ* is the angle between the mirror and the laser beam. Erasure of the SRGs was done with 530 nm LED and beam was focused directly on SRG topography with intensity of 100 mWcm^−2^. The inscription and erasure of the SRGs was monitored with a low‐power (1 mW) 633 nm He–Ne laser and the diffraction efficiency of the first order diffracted beam was measured.

##### Silicon Master and PDMS Replica Fabrication

Micropatterned silicon master was fabricated using Ø100 mm silicon wafer (University Wafer). This was rinsed with isopropyl alcohol (IPA) and water and dried with pressurized nitrogen. The wafer was then oxidized with plasma for 7 min at 200 W. GM1020 (Gersteltec) resist was spin‐coated to obtain 350 nm groove depth. The spin‐coated wafer was left for 10 min at room temperature for relaxation. The wafer was pre‐baked first at 65 °C for 5 min and then at 95 °C for 15 min. Exposure through photomask (1 μm wide microgrooves, equally spaced) was done with 20 mWcm^−2^ for 18 s (exposure dose = 360 mJcm^−2^). The wafer was left for 10 min at room temperature and post‐baked at 65 °C for 10 min and at 95 °C for 60 min. The wafer was developed in mr‐Dev 600 (micro resist technology GmbH) for 1 min 30 s and rinsed with isopropyl alcohol and water and dried with pressurized nitrogen. Last the wafer was hard‐baked at 150 °C for 15 min. To increase the lifetime of the fabricated silicon master, micropatterned PDMS replicas were fabricated. A mixture of PDMS precursors was prepared as reported above. The solution was poured on patterned silicon master, after which degassing of the mixture was performed in a vacuum chamber for 1 h. PDMS was cured in the oven at 65 °C for 1 h. The PDMS was carefully cut and peeled from the silicon master. The produced PDMS replicas were hydrophilized with trichloro‐silane (tridecafluoro‐1,1,2,2‐tetrahydrooctyl, Sigma‐Aldrich). Trichloro‐silane (35 μl) was placed in a petri dish and PDMS replicas were placed next to the drop. PDMS replicas were incubated with trichloro‐silane for 4 h in a vacuum chamber.

##### Microcontact Printing (μCP)

Micropatterned PDMS stamps were produced as follows. A mixture of PDMS precursors was prepared as reported above. The solution was poured on replicas, after which degassing of the mixture was performed in a vacuum chamber for 1 h. PDMS was cured in the oven at 65 °C for 1 h. The stamps were carefully cut and peeled from the master. Flat stamps were fabricated by pouring the prepolymer PDMS on a flat silicon wafer and used directly. PDMS stamps were treated with oxygen plasma (Pico, Diener electronic GmbH) for 27 s using 50 W power and 0.3 mbar pressure. Right after this, the PDMS stamps were incubated with either 50 μgml^−1^ FITC‐conjugated gelatin (AnaSpec) in Milli‐Q water or 50 μgml^−1^ monomeric rat tail type I collagen (Thermo Fischer Scientific) in 0.02 N acetic acid for 40 min. Before stamping, the samples were treated with oxygen plasma using the same parameters. The excess protein was removed from the stamp by washing three times with phosphate buffered saline (PBS) and once with Milli‐Q water. Stamps were dried under nitrogen gas flow and brought into contact with the plasma treated samples for 2 min. Pressure was gently applied with tweezers and a small 5 g weight was placed on top of the stamps to help the complete pattern transfer. The stamp was gently removed, gelatin printed samples were stored dry whereas collagen printed samples were covered with PBS and stored at 4 °C. Before cell seeding, the collagen patterned samples were passivated to block attachment of cells to nonpatterned areas. This was done by incubating the samples with 0.03% gml^−1^ Pluronic F‐127 (Sigma‐Alrdich) in Milli‐Q water for 1 h, after which the samples were washed with PBS.

##### Cell Culture

Epithelial Madin–Darby canine kidney type II (MDCK II) cells were used for this study. They were cultured at 37 °C under a humidified atmosphere with 5% CO_2_ in a culture medium consisting of MEM GlutaMax (Gibco) supplemented with fetal bovine serum (10%) and penicillin/streptomycin (1%). Before cell seeding, the samples were sterilized under UV light for 40 min. The nonmicrocontact printed samples were coated with 50 μgml^−1^ monomeric rat tail type I collagen solution (Thermo Fischer Scientific) in 0.02 N acetic acid for 40 min.

##### Immunolabeling

Cells were fixed with 4% paraformaldehyde for 10 min, washed with PBS, permeabilized for 10 min with permeabilization buffer (0.5% BSA, 0.5% Triton‐X 100 in PBS) and blocked for 1 h using 3% bovine serum albumin in PBS. The samples were labeled with rabbit anti‐pFAK (1:200, Abcam, #ab81298) and rat anti‐Uvomorulin/E‐Cadherin (1:100, Sigma‐Aldrich). Secondary antibodies used were anti‐rat‐Alexa 568 (1:200, Thermo Fisher Scientific #A110077) and anti‐rabbit‐Alexa 647 (1:200, Thermo Fisher Scientific, #A21244). Actin cytoskeleton was labeled using 488‐phalloidin (1:50, Sigma‐Aldrich #49 409). Samples were mounted with ProLong Diamond antifade mountant with 4′,6‐diamidino‐2‐phenylindole (DAPI) (Thermo‐Fisher Scientific, #P36935), which stains the cell nuclei.

##### Optical Imaging

Samples were imaged with an optical (Zeiss) and confocal microscope (Nikon A1R laser scanning confocal microscope, Nikon Instruments Europe BV). For confocal microscopy the laser lines used were 405, 488, 561, and 633 nm. For each image, the laser intensity was adjusted to avoid photobleaching, and detector sensitivity was tuned to optimize the image brightness. A 60×/1.4 Plan‐Apochromat oil immersion objective and 20×/0.8 Plan‐Apochromat air immersion objective were used to capture 1024 × 1024 pixel images. The data was in the form of 3D z‐stacks, which included 30‐40 slices each with 150–250 nm interval. Time lapse microscopy was performed with EVOS FL auto (Thermo Fisher Scientific).

##### Topography Erasure with Confocal Microscope

The SRG topography was erased with a LSM780 laser scanning confocal microscope (Zeiss). Plan‐Apochromat ×20/1.4 water immersion objective was used during erasure. The samples were either in dry, liquid or cell culture environment during the irradiation. The samples were illuminated for 5 min with a fluorescent lamp filtered in the blue region (470 ± 40 nm) with intensity of 1.5 Wcm^−2^. Bright field images of the topography were captured before and after erasure. With MDCK II cells, the samples were irradiated with the fluorescent lamp, after which the cells were detached from the sample with trypsin for surface characterization or fixed after 2 h for immunolabeling.

##### Live/Dead Viability Assay

MDCK II cells were seeded on photopatterned bilayer and cultured on top of the samples for 24 h. Topography was erased as described above. After 3 h from the erasure, cells were washed with PBS and stained using LIVE/DEAD Viability/Cytotoxicity Kit *for mammalian cells* (Thermo Fischer Scientific) by adding 600 μl LIVE/DEAD reagent solution on each sample, containing 0,50 μl/ml calcein AM and 2 μl/ml ethidium monodimer‐1 in PBS. The samples were incubated at 37 ºC under a humidified atmosphere with 5% CO_2_ for 30 min. Following incubation, reagent solution was aspired and 600 μl PBS was added to prevent the cells from drying. The samples were imaged with a confocal microscope (Nikon A1R laser scanning confocal microscope) using 488 nm and 561 nm laser lines. A 20×/0.8 Plan‐Apochromat air immersion objective was used to capture 1024 × 1024 pixel images.

##### Image and Statistical Analyses

The distribution of focal adhesions was analyzed with fast Fourier transform of the focal adhesion channel using FFT plugin in ImageJ. Prior to generating FFT image, circular region of 900 pixels was cropped and the FFT image was generated from this region. ImageJ was used to measure the elongation, area and orientation of focal adhesions. Further analysis of focal adhesion elongation and orientation was done with MomentMacroJ v1.4B script (https://www.hopkinsmedicine.org/fae/mmacro.html). The graphs in Figure 3d–f represent average of 100 quantified focal adhesions from 10 separate images. In Figure 5c,d and Figure 7c the graphs represent average of 30 quantified focal adhesions from 2 separate images. Prior to analysis, the focal adhesion images were processed to remove pixel noise as described by Maruoka et al.^[^ ^68^ ^]^ and Ventre et al.^[^ ^69^ ^]^ The principal moments of inertia were measured (i.e., maximum and minimum) and the cell elongation was defined as the ratio of these values (maximum/minimum). Higher values indicate more elongated focal adhesions. The orientation of the focal adhesion was defined as the angle between the surface pattern direction and the maximum axis. Statistical analyses were done with Origin, version 2019b (OriginLab Corporation) and MATLAB. We estimated the statistical power of the test for experiments which had less than 100 hundred focal adhesion quantified. We estimated that the statistical differences were significant with actual power values over 75%. Our data was found to have a nonnormal distribution, thus nonparametric Kruskal–Wallis test with Bonferroni and Dunn‐Sidak post hoc tests were used to evaluate the statistical significance.

## Conflict of Interests

The authors declare no conflict of interests.

## Data Availability Statement

Data available on request from the authors.

## Supporting information

Supplementary Material
