# Supplementary material for: Light‐Responsive Bilayer Cell Culture Platform for Reversible Cell Guidance
Source: Small Sci. 2021 Dec 16;2(3):2100099. doi: 10.1002/smsc.202100099 (PMC11935986; doi:10.1002/smsc.202100099)
Supplement: Supplementary file 1 — Supplementary Material [file SMSC-2-2100099-s001.zip › renamed_c7228.pdf]

Supporting Information

Light-responsive bilayer cell culture platform for reversible cell guidance

Mari Isomäki, Chiara Fedele, Lotta Kääriäinen, Elina Mäntylä, Soile Nymark. Teemu O. Ihalainen, Arri Priimagi**

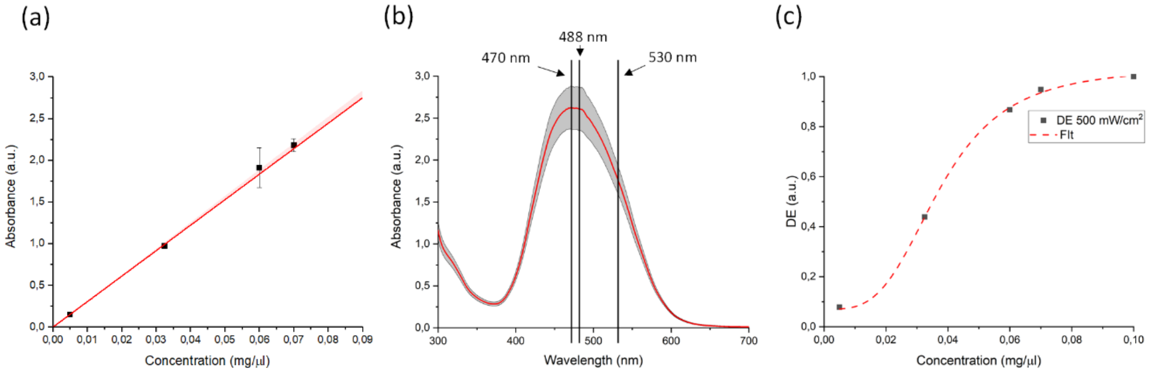

Figure S1. a) UV/Vis spectrophotometry of different concentrations of spin coated DR1g solutions. The absorbance values were taken at 480 nm. The red curve is the linear fitting curve (fitted range between experimental points and extrapolated for the external points). **b)** UV/Vis spectra of 5 different spin coated DR1-glass thin films (9 % w/v in $CHCl_3$). The red curve is the average of all the 5 samples and the gray contour area represents the error. The vertical lines represent the wavelengths used for erasure with confocal lamp (470 nm), SRG inscription (480 nm) and erasure with LED (530 nm). **c)** Normalized diffraction efficiency (DE) maximum values (black squares) after 10 min irradiation at Lloyd's mirror with 500 mWcm^{-2} 488 nm laser interference patternindicating that abovt ca. 8%, the DE does not significantly depend on the solution concentration, hence on the thickness of the samples.

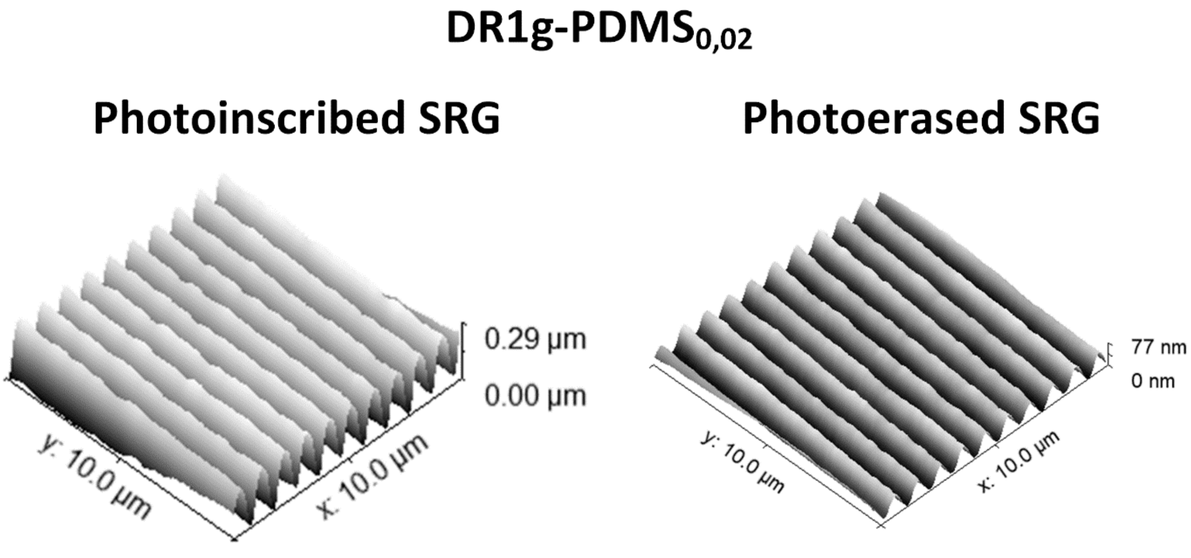

Figure S2. AFM image of DR1g-PDMS$_{0,02}$ surface after SRG inscription with intensity of 500 mWcm^{-2} and SRG erasure with 530 nm LED.

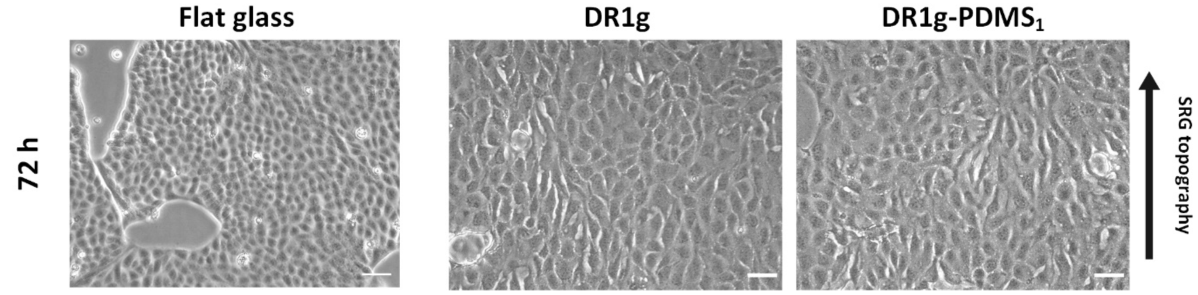

Figure S3. Optical microscope images of MDCK II cells on a flat glass control, surface patterned DR1g film and DR1g-PDMS1 bilayer after 72 h from cell seeding. At this timepoint cells were forming a confluent cell layer. Black arrow indicates the SRG topography direction. Scale bars: 50 µm.

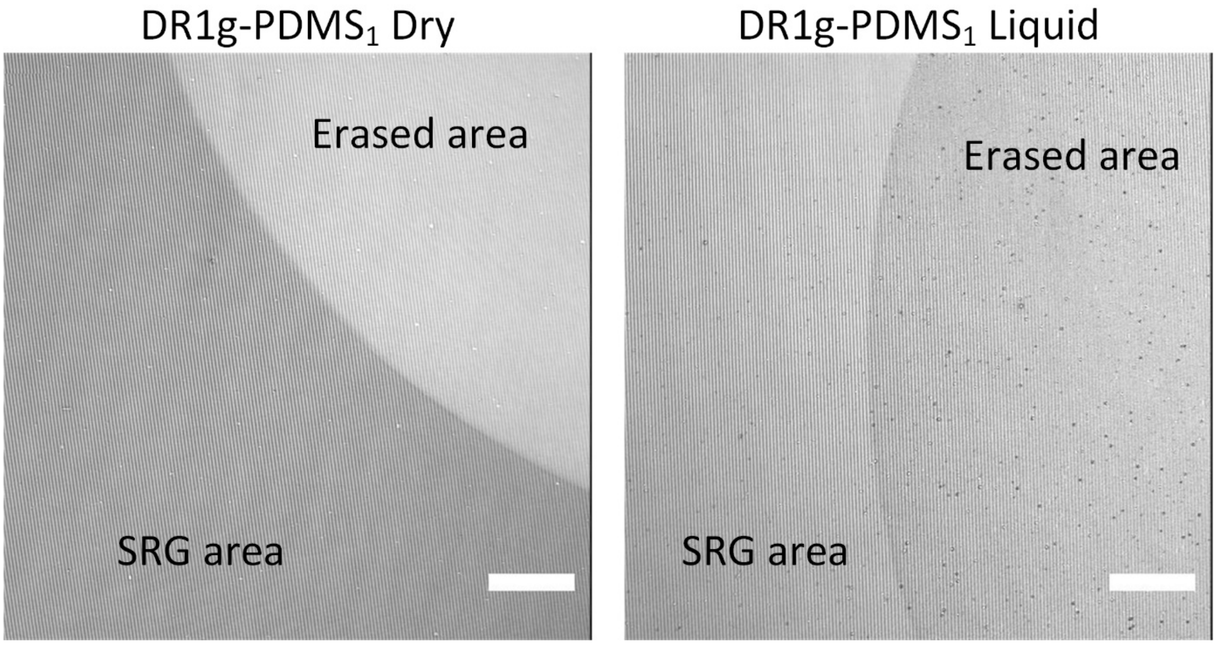

Figure S4. Bright-field image between SRG and erased area of DR1g-PDMS$_1$ bilayer after erasure with a fluorescent lamp of a confocal microscope (irradiation time 5 min) in dry and liquid environment. The process resulted in clearly visible round erased area. Scale bars: 50 µm.

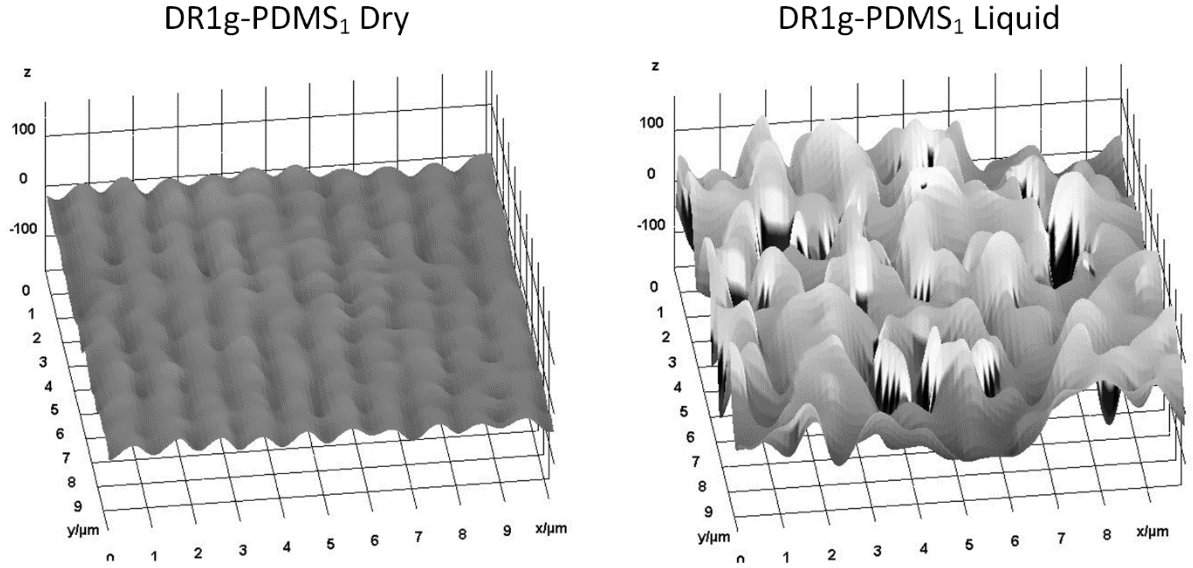

Figure S5. 3D reconstructions of DHM images of erased SRG topography on DR1g-PDMS$_1$ bilayer in dry and liquid environment, when topography was erased with a fluorescent lamp of a confocal microscope (irradiation time 5 min). Surface erasure in the presence of liquid resulted in increased roughness of the surface.

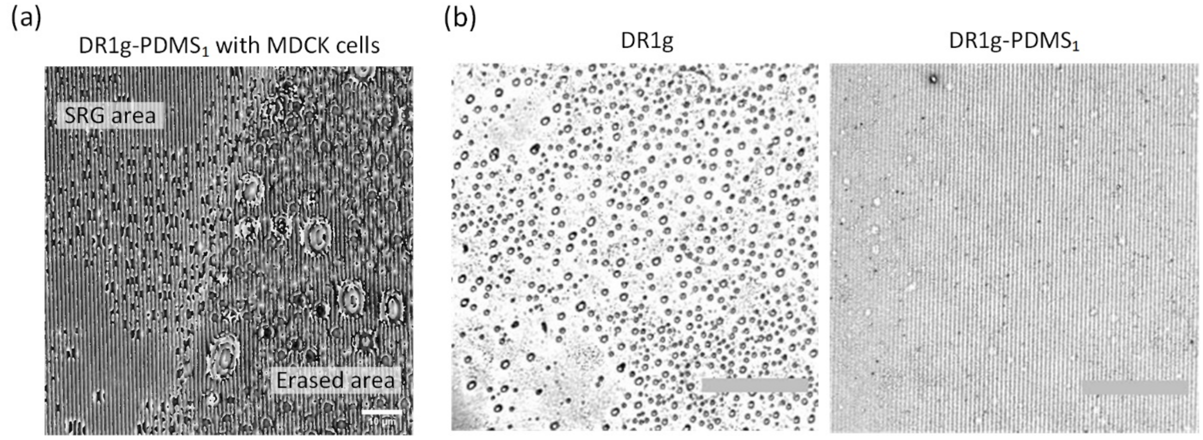

Figure S6. a) DHM image between SRG and erased area of DR1g-PDMS$_1$ bilayer, when topography was erased with a fluorescent lamp of a confocal microscope (irradiation time 5 min) in cell culturing environment with MDCK II cells. The topography erasure was conducted with a fluorescent lamp of a confocal microscope for 5 min. This resulted in visible erased area. Scale bar: 10 µm. b) Bright-field image of erased area of DR1g and DR1g-PDMS$_1$ bilayer in cell culture environment. Scale bars: 50 µm.

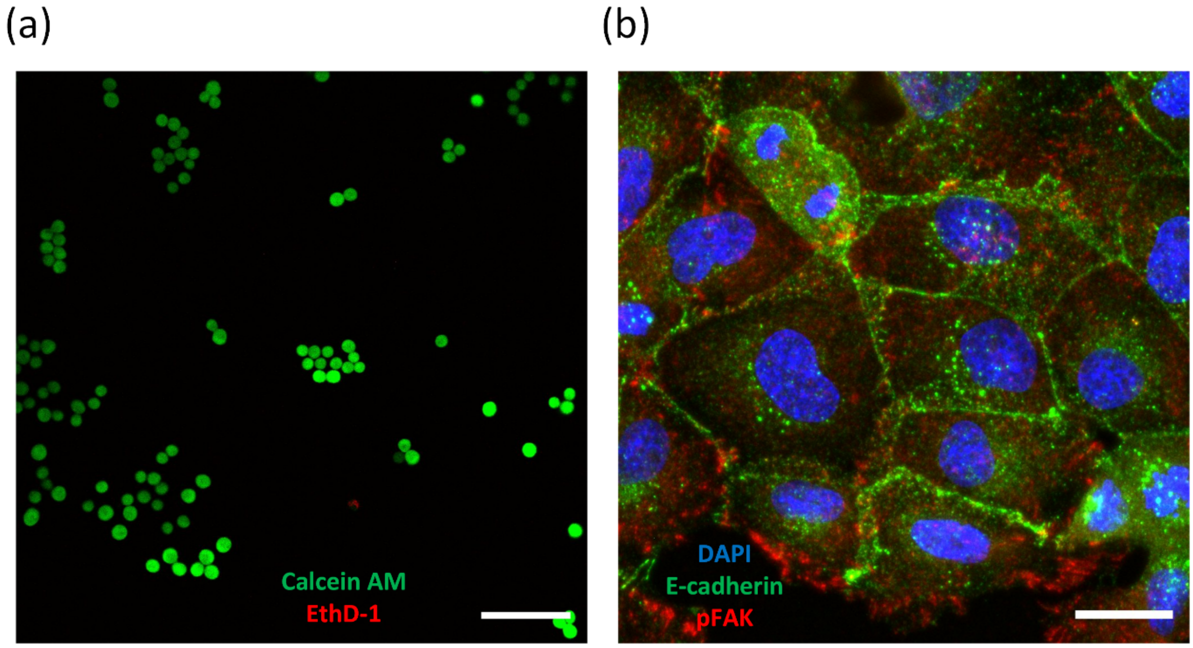

Figure S7. a) Live/Dead viability assay image from areas that are not illuminated with confocal fluorescent lamp. MDCK II cells were seeded on samples in which DR1g was spin coated to the bottom side of the glass coverslip and glass substrate was at cell-material interface. Live cells are indicated as a green fluorescent signal (calcein AM) and dead cells as red fluorescent signal (EthD-1). Scale bar: 100 µm. b) Immunolabeled MDCK II cells on phototoxicity control samples. Control samples were prepared by spin coating DR1-glass on the bottom side on a glass coverslip (to ensure similar light attenuation as in the case of photopatternable samples) and 1 wt% PDMS on the top side of the same coverslip. MDCK II cells were seeded on the PDMS side on the coverslip. After 24 h cells were illuminated with a fluorescent lamp of a confocal microscope (filtered in the blue region (470±40 nm) of the visible spectrum for 5 min. Cells were fixed after 2 h from irradiation and immunolabeled. Labels used were DAPI (chromatin), E-cadherin (cell-cell junctions) and pFAK (mature focal adhesions). Scale bar: 20 µm.

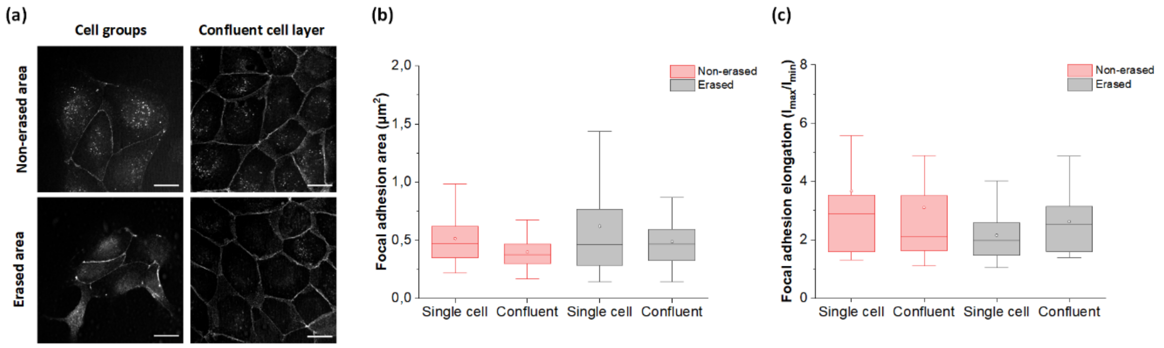

Figure S8. a) Immunolabeled MDCK II cells on DR1g-PDMS$_1$ bilayer samples on non-erased (top) and erased (bottom) SRG topography in the presence of small cell groups (left) and confluent cell layer (right). Erasure was conducted by illuminating the samples with a fluorescent lamp of a confocal microscope filtered in the blue region (470±40 nm) for 5 min. Label shown here is E-cadherin (cell-cell junctions). Scale bars: 20 µm. c) Focal adhesion b) area and a) elongation when topography was erased in the presence of small cell groups and confluent cell layer. The graphs represent average of 30 quantified focal adhesions from 2 separate images.

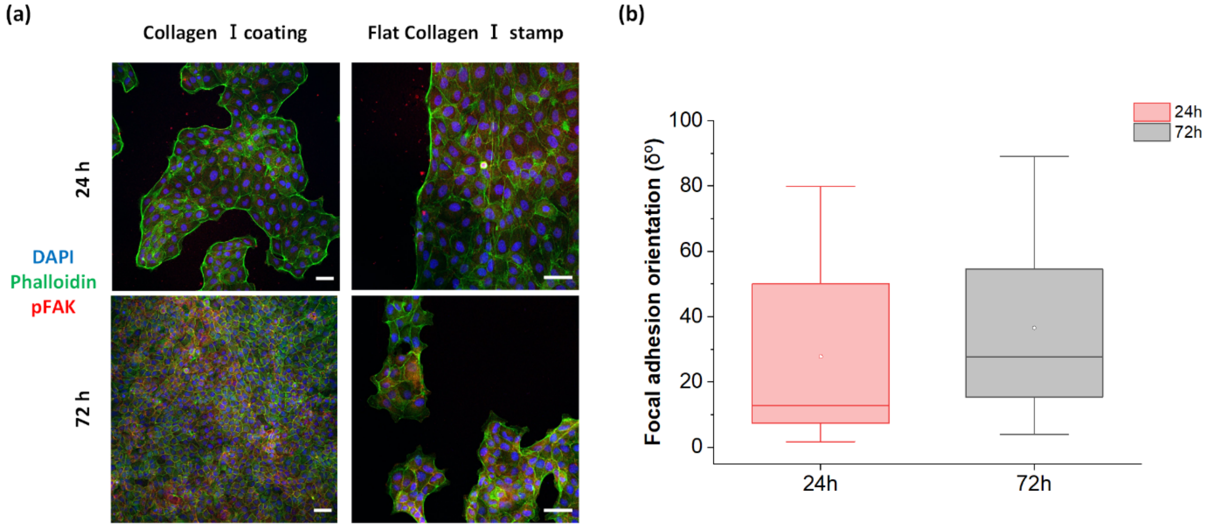

Figure S9. a) Immunolabeled MDCK II cells on collagen I-coated DR1g-PDMS1 bilayer, which has been patterned with flat collagen I stamp, at different time points (24 h, 72 h). The labels used were chromatin (DAPI stain, blue), actin (phalloidin stain, green) and pFAK (red). Scale bars: 50 μm. b) Focal adhesion orientation on protein patterned samples after 24 h and 72 h. The graphs represent average of 30 quantified focal adhesions from 2 separate images.

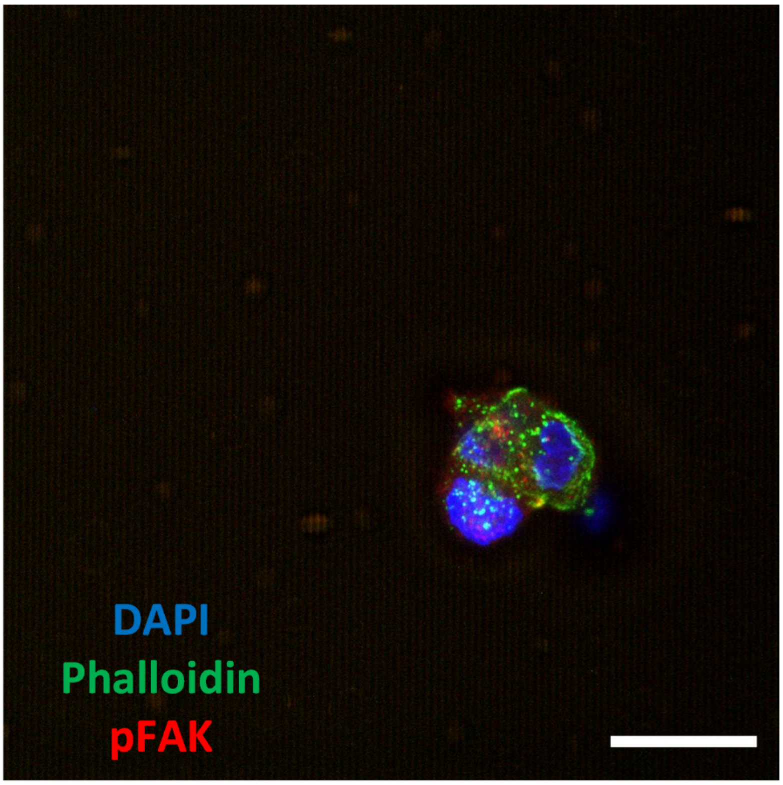

Figure S10. Immunolabeled MDCK II cells on DR1g-PDMS1 bilayer on area that is passivated with Pluronic F-127. Labels used were chromatin (DAPI stain, blue), actin (phalloidin stain, green) and pFAK (red). Only few cells were seen on the passivated area. Scale bar: 20 μm.
